# A case control study of the factors associated with occurrence of aerodigestive foreign bodies in children in a regional referral hospital in South Western Uganda

**DOI:** 10.1186/s12901-016-0026-4

**Published:** 2016-03-15

**Authors:** Doreen Nakku, Richard Byaruhanga, Francis Bajunirwe, Imelda T. Kyamwanga

**Affiliations:** Department of ENT Surgery, Mbarara University of Science and Technology, P.O Box 1410, Mbarara, Uganda; ENT Department 5th Floor, New Mulago Hospital Complex, Makerere University College of Health Sciences School of Medicine, Kampala, Uganda; Department of Community Health, Mbarara University of Science and Technology, P.O Box 1410, Mbarara, Uganda

**Keywords:** Aerodigestive, Foreign bodies, Factors, Occurrence, Uganda

## Abstract

**Background:**

Aerodigestive foreign bodies (ADFB) in children are a common emergency in ENT clinics globally. The aim of this study was to determine the prevalence and common types of ADFB’s presenting to a referral hospital in South Western Uganda, and to review clinical presentation and factors that influence their occurrence among children under 12 years of age.

**Methods:**

We conducted a case control study comprising 40 cases and 80 unmatched controls. Consecutive and random sampling were used for the cases and controls respectively. A questionnaire was used to collect data. Clinic records were reviewed to calculate prevalence.

**Results:**

Prevalence was 6.6 % of all paediatric cases seen in the ENT department that year. The most common symptoms included: history of choking [45 %], sudden cough [72.5 %], stridor [60 %] and failure to swallow [35 %]. The most common location for an airway foreign body was the right main bronchus [40 %] and the upper one third of the oesophagus [32.5 %] for digestive tract foreign bodies. Seeds and coins were most frequently removed. Children from upper level SES had a significantly lower risk of foreign body occurrence [*OR = 0.29, p = 0.02*] compared to those from a low SES. Also significantly, most cases were referrals from other government health centres [*p =* <0.01]. The male to female ratio among cases was 2:1. Children of older mothers were less likely to have an ADFB.

**Conclusion:**

Prevalence of ADFB’s is relatively high. The most common symptoms are a history of choking, cough and failure to swallow. Age under 5 years, male sex, younger maternal age and low socioeconomic status increased odds of ADFBs.

## Background

Worldwide, aerodigestive foreign bodies [ADFBs] are a common emergency in Ear, Nose and Throat [ENT] clinics and remain one of the main causes of morbidity and mortality especially in children under 3 years [1, 2]. Despite public awareness, and improvements in medical care, worldwide annual incidence of deaths from foreign body aspiration, is estimated to be between 500 and 2000 [3, 4].

The disease burden of ADFB is not known but varies according to geographic location. Anecdotal evidence suggests ADFB is one of the most common emergencies seen at the ENT Department at Mbarara Regional Referral Hospital [MRRH]. Little is documented regarding its prevalence and the characteristics of the children presenting with ADFBs. This lack of documentation may contribute to delays in diagnosis and inadequate management, and makes prevention through education of parents difficult. Determining these factors would aid timely diagnosis and management and provide data to inform health education and other primary prevention interventions for ADFB.

Therefore this study was undertaken to determine the proportion of ADFBs among all paediatric cases seen in the ENT clinic at MRRH, their clinical characteristics and factors associated with the condition.

## Methods

### Study design and population

We conducted a case control study at the ENT Clinic at MRRH, a tertiary referral facility and teaching hospital in Mbarara Municipality, South Western Uganda. On average the clinic receives approximately 300 outpatient visits per month including children and adults. The catchment area for the hospital consists of 11 districts each with a total population of about 3–4 million people.

The study population comprised children aged between 6 months and 12 years who presented with various ENT related complaints.

### Selection of cases and controls

The cases were defined as children who had aspirated or ingested a foreign body. The diagnosis was confirmed either by imaging, or at bronchoscopy and esophagoscopy. Only cases with a confirmed diagnosis were enrolled. Children who presented to the ENT clinic with other symptoms not related to aero-digestive foreign bodies were used as controls and were randomly selected from a group of eligible patients by drawing lots. For every case, two controls were selected to acquire data regarding family demographics, source of referral and socioeconomic status, for comparison with our study group. Frequency matching for age was done to ensure equal distribution of age among the cases and controls. Sample size calculation was done using the Kelsey [5] formula giving a total sample size of 40 cases and 80 controls to give a total of 120 children.

### Exclusion criteria

The study excluded children 6 months to 12 years of age with anatomical or physiological abnormalities that might interfere with normal feeding and airway protection. Examples of conditions warranting exclusion were patients with cleft lip and palate, diffuse esophageal spasm and tracheo-esophageal fistula, esophageal strictures, those who had recently had bowel surgery and children with mental retardation or neurological abnormalities.

### Data collection

This study was conducted over a period of 6 months between January and June 2013. Parents or guardians provided a written consent to include their children in the study. Thereafter they were interviewed using a questionnaire we developed and found to be reliable to administer and easy to understand, to establish the circumstances surrounding the aspiration or ingestion of the foreign body, and any interventions that were performed at home. Socioeconomic status was measured by adopting and modifying the Kuppuswammy scale for socioeconomic status that has been used previously in India and in Nepal [6].

In order to calculate the prevalence of ADFB over one year, we reviewed hospital ENT clinic records for the time frame between July 2011 and June 2012. Prevalence was calculated as number of cases divided by total number of ENT patients aged 6 months and 12 years. Cases where children were younger than 6 months or older than 12 years were not considered for the calculation. Moreover, none of the cases used in calculating prevalence were considered for any further analysis.

### Data analysis

STATA 11.0 was used for statistical analysis. Prevalence of ADFBs was calculated by dividing cases of ADFB a percentage of total number of children seen in the clinic between July 2011 and June 2012. Frequencies and percentages described the common types of foreign bodies and characteristics of the patients. Logistic regression analysis was used to assess factors associated with the occurrence of ADFBs and odds ratios were calculated. The level of significance was set at p < 0.05.

### Quality control and ethics

The proposal was submitted and approved by the Faculty of Medicine Research Ethics Committee and the Mbarara University Institutional Review Board. The parents or guardians provided consent for their children to participate in the study and where older children were involved, assent was sought. All children with ADFB received the expected standard of care and it was made clear to the parents and guardians that refusal to participate in the study would not in any way interfere with the clinical management they were entitled to. Clinical examinations were carried out by the principle investigator who was also present in theatre for all bronchoscopy and esophagoscopy procedures. Radiological films, when available, were reviewed by the hospital radiologist to provide advice on management approach.

## Results

In the review of records, there was a total of 64 cases of pediatric ADFB out of 971 pediatric cases treated in the ENT clinic between July 2011 and June 2012 yielding a prevalence of 6.6 %. Of the 64 cases, 36 [56.3 %] were male.

**Fig. 1:**
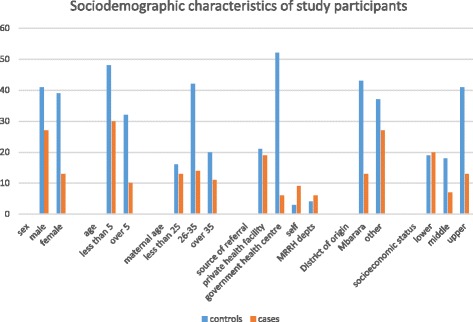
A graph summarizing the sociodemographic characteristics of the study participants

The socio-demographic characteristics of all study participants are illustrated in Fig. 1. Children under 5 years of age accounted for 75 % of the cases and of these cases, there were twice as many males as females [Male: Female ratio of 2:1].

Over two thirds of the controls were referred from Government health centres, while majority of the cases [47.5 %] were referred from different private facilities. This result was significant with *p* = <0.01. Only six patients were referred to the clinic from other departments within the hospital; predominantly from the Pediatrics ward and the Children’s Out Patient’s Department.

The most frequent symptoms reported are shown in Table 1, and included sudden onset of cough [72 %] and difficulty in breathing [60 %] which are associated with aspiration of a foreign body into the airway and vomiting and retching [42.5 %] which are associated with swallowing of a foreign body. Only one parent/guardian attempted a Heimlich manoeuvre after witnessing the child swallowing a coin. Approximately 30 % of the children presented with fever , suggestive of possible secondary infection. Stridor [45 %], a hoarse voice [40 %], reduced chest movement and chest recessions [52.5 %] are common findings in foreign body aspiration and were common symptoms among the cases as shown in Table 2.

**Table 1:** Symptoms of children with aerodigestive foreign bodies and home interventions attempted

		Frequency	Percentage
Symptom	Sudden onset of cough	29	72.5
	Sudden onset difficulty in breathing	24	60.0
	Noisy breathing	16	40.0
	Change in colour to blue [cyanosis]	3	7.5
	Hoarseness of the voice	13	32.5
	Vomiting and retching	17	42.5
	Drooling of saliva	11	27.5
	Failure to swallow feeds	14	35.0
Intervention at home	Finger sweep	12	30.0
	Induced vomiting	4	10.0
	Gave local herbs	6	15.0
	Heimlich manoeuvre	1	2.5
	No intervention	19	47.5

**Table 2:** Examination Findings of Cases

Presenting complaints		Frequency	Percentage
Temperature	Normal range	28	70.0
	Low grade fever	6	15.0
	High grade fever	6	15.0
Stridor	Yes	18	45.0
	No	22	55.0
Chest movement on breathing	Normal	22	55.0
	Reduced chest movement on left side	4	10.0
	Reduced chest movement on right side	11	27.5
	Reduced chest movement on both side	3	7.5
Chest recessions	Yes	21	52.5
	No	19	47.5
Air entry into the lungs	Normal both sides	14	35.0
	Reduced on left side	7	17.5
	Reduced on right side	16	40.0
	Reduced on both sides	3	7.5
Voice of the child	Normal	24	60.0
	Hoarse	16	40.0
X-ray findings	Normal x-ray	1	3.85
	Collapsed lung	4	15.38
	Heterogeneous opacity	2	7.69
	Foreign body seen on x-ray	19	73.08

**Fig. 2:**
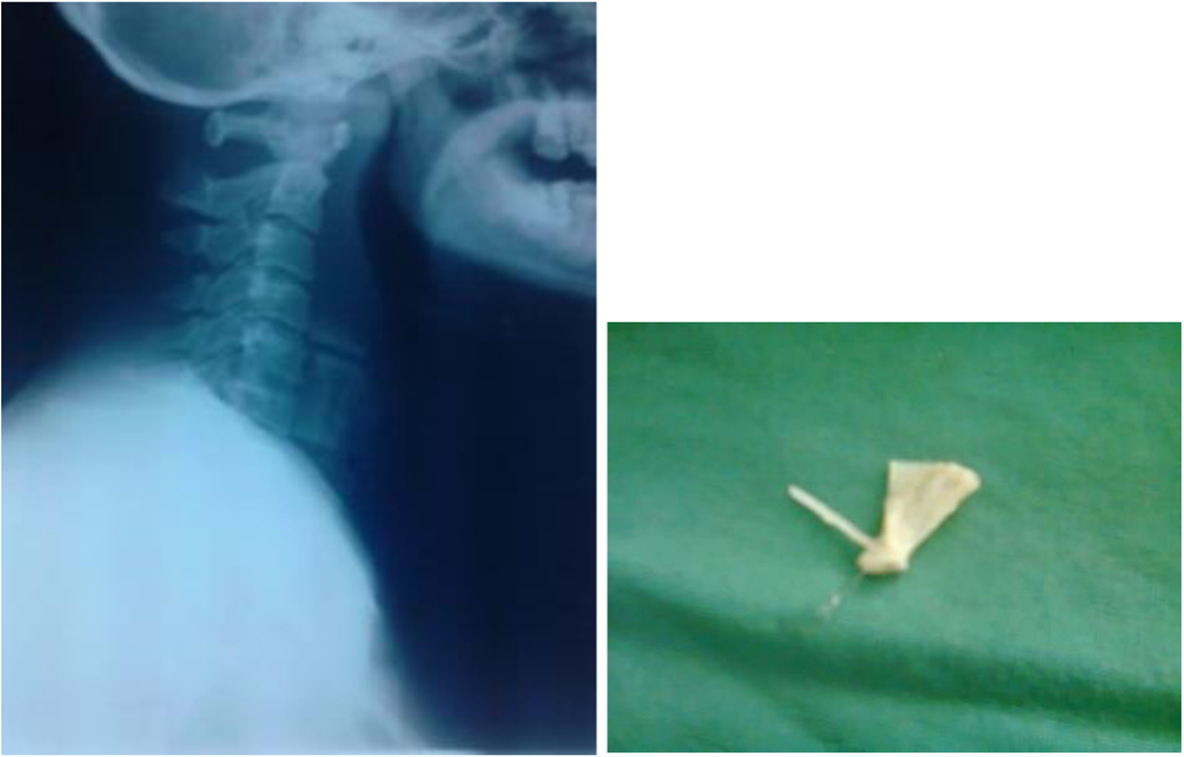
Foreign body in the esophagus (left), fish bone removed from airway (right)

Out of the 26 X-rays that were received during the study, 73.1 % [*n* = 19] confirmed the presence of a radiopaque foreign body in the aerodigestive system (Fig. 2) while 23.1 % [*n* = 6] showed heterogeneous opacity or lung collapse which are both possible effects of a foreign body.

At least 60 % of the foreign bodies in the airway were removed from the right main bronchus and an example of such a foreign body is shown above in Fig. 2 on the right. In the oesophagus almost all of them [*n* = 14 or 92.8 %] were removed from the upper third at the level of the cricopharyngeal sphincter.

As shown in Table 3, equal numbers of organic and inorganic foreign bodies were removed or identified. Coins accounted for 55 % of all inorganic foreign bodies and seeds accounted for 80 % of the organic foreign bodies. 95 % of all foreign bodies were successfully removed by bronchosopy or oesophagoscopy. Two of the cases presented with airway foreign bodies [a ball bearing and cylindrical metallic casing of a pencil eraser] which were identified on radiography and by bronchoscopy but removal was unsuccessful via endoscopic methods; these children were referred for thoracotomy.

**Table 3:** Type of foreign body removed and point of removal from the aerodigestive tract

	Frequency	Percentage
Point of removal		
Trachea	6	15.0
Left bronchus	4	10.0
Right bronchus	16	40.0
Oesophagus upper 1/3	13	32.5
Oesophagus mid 1/3	1	2.5
Type of foreign body		
Organic	20	50.0
Seeds [beans, groundnuts, coffee, maize]	16	40.0
Foods [avocado, fish bone, egg shell]	4	10.0
Inorganic	20	50.0
Ugandan shillings coins	11	27.5
Plastics [pen tips]	2	5.0
Metals [ball bearing, metallic button, soda bottle top, torch bulb, metallic eraser casing]	7	17.5

### Factors associated with ADFB

Table 4 shows the factors associated with ADFBs. The risk of sustaining an ADFB was found to be statistically higher for children from districts outside of Mbarara [*OR = 2.41, p = 0.*03]. Therefore 67.5 % of cases were from districts outside Mbarara [*p = 0.02*]. Children from families of upper level SES had significantly lower odds of suffering an ADFB when compared to those from a lower socioeconomic status, [*OR = 0.30, p = 0.01*]. Male children are twice as likely to present with an ADFB compared to females. Older maternal age is protective; children of younger mothers were more at risk of sustaining an ADFB. When the risk of a child sustaining an ADFB was adjusted for source of referral and SES, the regression model in Table 5 demonstrated that upper level SES and referrals from government health centres were significant factors with *p = 0.02* and *p = <0.01* respectively.

**Table 4:** Relationship between the non-clinical characteristics and the risk of a child sustaining an aerodigestive foreign bodies

Risk factor		Odds Ratio	95 % Confidence Interval	p-value
Age of child [years]	≤5	1		
	>5	0.5	0.21 – 1.16	0.11
Sex	Male	1		
	Female	0.51	0.23 – 1.12	0.09
Age of mother [years]	≤25	1		
	26 – 35	0.41	0.16 – 1.06	0.07
	>35	0.67	0.24 – 1.91	0.46
Socioeconomic status	Lower	1		
	Middle	0.37	0.13 – 1.08	0.07
	Upper	0.30	0.12 – 0.73	0.01*
District of origin	Mbarara	1		
	Other	2.41	1.09 – 5.34	0.03*

**Table 5:** Regression model showing risk of sustaining an aerodigestive foreign body adjusted for source of referral and socioeconomic Status

Characteristic		Odds Ratio	95 % Confidence Interval	p-value
Source of referral	Private health facility	1		
	Government health centre	0.11	0.04 – 0.34	<0.01*
	Self	2.89	0.63 – 13.2	0.17
	MRRH departments	2.69	0.46 – 15.78	0.27
Socioeconomic status	Lower	1		
	Middle	0.53	0.15 – 1.90	0.33
	Upper	0.29	0.1 – 0.84	0.02*

## Discussion

Foreign bodies in the aerodigestive tract are a major source of morbidity in developing countries, and understanding the features that predispose patients to the risk aspirating or swallowing a foreign body is critical to diagnosis, treatment, and prevention. As a first step toward improving overall management of this problem, we catalogued the prevalence of ADFB amongst pediatric patients at a tertiary care and academic medical center in Uganda, and identified potential risk factors.

The prevalence of ADFBs varies depending on the region and the hospital, and sometimes the two proportions are variable. For example, the University of Port Harcourt Teaching Hospital in Nigeria recorded a prevalence of laryngeal foreign bodies of 2.5 % without considering oesophageal foreign bodies [7]. In Mulago National Referral Hospital in Uganda, ingested foreign bodies are a common occurrence in the ENT department though the actual prevalence is unknown [8]. A retrospective review of records from the same hospital between 1993 and 1999, reported 240 bronchoscopies performed to remove aspirated foreign bodies which averages approximately 40 bronchoscopies per year [Awubwa, Unpublished observation]. Therefore, a prevalence of 6.6 % at MRRH is high, and may be explained by the fact that MRRH serves a much larger population than its catchment area as it is the only regional referral hospital in South Western Uganda providing ENT services. Some patients are referred from as far as the Democratic Republic of Congo, Tanzania, Rwanda and other in-country regions. In addition, MRRH is a government hospital providing free services, which contributes to the large numbers of patients seen.

When dealing with a patient presenting with an aerodigestive foreign body, a high index of suspicion is required to make a diagnosis. The children who had aspirated or swallowed foreign bodies presented with various but related symptoms and signs. The most common symptoms were sudden onset of cough [72.5 %] and dyspnea [60 %]. Noisy breathing also referred to as stridor was only seen in 40 % of the cases. These symptoms occur because on aspiration, the foreign body causes mucosal irritation in the airways prompting the cough reflex. The total or partial obstruction of the main airways from the aspirated object leads to increased velocity of the air as it passes  through a narrowed lumen during respiration, hence the noisy breathing. A swallowed foreign body in the oesophagus can at times be big enough to push against the trachea and cause stridor [9]; but this scenario was not encountered in this study. These findings are in agreement with others that noted that following foreign body aspiration in children, a high degree of suspicion was essential in arriving at the diagnosis and that the common symptoms were sudden onset of cough, dyspnea with noisy breathing in majority of the cases [10].

Radiography, though important, was much more useful when the foreign body was radiopaque so it could be identified on the film or if there was a suspected complication that could be identified on the film such as lung collapse. Digoy [11] also concluded that radiography in aerodigestive foreign bodies is much more important when dealing with esophageal foreign bodies than airway foreign bodies. In our study, there were only 2 radiopaque foreign bodies found in the airways- a ball bearing and a metallic casing of an eraser. However, as stated by Gilyowa [12], the absence of an identifiable foreign body on the radiograph does not rule out the presence of one in the system; therefore a high index of suspicion should always be maintained.

Our study demonstrated a high incidence of cervical esophageal foreign bodies as expected. This location [the cricopharyngeal sphincter] is the first of three anatomical narrowings of the oesophagus. Any large object is likely to fail to negotiate this section and instead get lodged causing the symptoms of drooling, retching and discomfort in the throat. For our aspirated foreign bodies, bronchoscopy usually revealed a foreign body in the right main bronchus, also similar to other literature reports. Studies done elsewhere also reported very similar results and explained this observation on the basis of the fact that the right main bronchus in children is more vertical and wider than the left [2, 13]. This anatomical feature makes it an easy target for a foreign body passing through the trachea. The most common foreign body removed from the esophagus was coins, particularly Sh. 100 coin. This is in agreement with the fact that worldwide, the most commonly swallowed foreign body is coins, which are a widely used legal tender [14].

During bronchoscopy for airway foreign body, seeds like groundnuts and beans were the most frequent foreign bodies removed. Similar to other areas in the world, they form a large part of the diet in the region so are easily accessible to children. This is comparable to studies by Ssewanyana [15] in Uganda, Black [16] in the United States and Mu [17] in China.

As in previous studies by Lima [18] and Ozdemir [19], in our study, foreign bodies were found to be much more common in children less than 5 years of age, peaking at about 3 years. This could be explained by the fact that in our community most mothers start weaning their babies at about 6 months of age by introducing solid foods. At this stage, the fact that the children’s dentition is not well developed to adequately chew the food and they cannot easily balance swallowing and breathing means that they are more prone to aspiration especially if food is not of the right consistency. The children also require more supervision as they eat and play especially when they are in the oral phase of development when they tend to place small objects in their mouths as also cited by a study conducted in Tanzania [12]. The risk of sustaining an ADFB reduces as the child gets older but is not completely eliminated.

In general, boys are considered more adventurous and curious compared to the girls as children grow. This may be the reason why a male: female ratio of 2:1 was found for the cases in our study. The regression model also showed this to be true and significant [*p = 0.03*]. This ratio is the same as that reported in other previous studies [12, 20, 21].

Looking at the socioeconomic status of the families from which all the children presented, majority of the controls were from the upper socioeconomic class while most cases were from the lower class. It was also observed that the higher the socioeconomic class of the family, the less the likelihood of the children sustaining an ADFB. This finding may be explained by the fact that families of middle or upper SES commonly have employed parents or guardians who can either afford to employ full time house help and child minders at home or to put their young children in professional day care facilities where there is always someone attending to them. Unlike parents from lower socioeconomic levels, these families rarely have more than 2 children under 5 years of age at the same time. This makes it easier to supervise and adequately care for the children. This is in agreement with the Turkish study by Ozdemir [19], which concluded that a higher social class is protective from ADFBs in children. In another study in from China, Mu [17] also found that there were slightly fewer foreign bodies among children from families living in urban areas [considered of a higher social class than those from the rural areas].

Significantly, we found that after adjusting for SES and the source of referral, the regression model showed that patients coming from other government health centres were more likely to have ADFBs when compared to those from private health facilities [*p = <0.01*]. The upper SES remained protective with the lowest risk of ADFB [*OR = 0.29, p = 0.02*]. This finding is likely because MRRH is the tertiary referral hospital for all other surrounding lower health centres and the only source of specialized health services in the region. The personnel at the lower level health centres are therefore more likely to refer to the tertiary centre any difficult cases.

## Conclusion and recommendations

Our findings suggest that male sex and poor socioeconomic status contribute to ADFB.

Educational initiatives designed to target these vulnerable populations may serve to decrease the incidence of ADFB in rural Uganda. Additionally, educating health workers to know the possible symptoms and signs of ADFBs will likely decrease morbidity and mortality from ADFB, by reducing delays in diagnosis.

## Abbreviations

ADFB: aerodigestive foreign body
ENT: ear, nose and throat
MRRH: Mbarara Regional Referral Hospital
SES: socioeconomic status
